# Supplementary material for: Global lake thermal regions shift under climate change
Source: Nat Commun. 2020 Mar 6;11:1232. doi: 10.1038/s41467-020-15108-z (PMC7060244; doi:10.1038/s41467-020-15108-z)
Supplement: Supplementary file 1 — Supplementary Information [file 41467_2020_15108_MOESM1_ESM.pdf]

Supplementary Information

Global lake thermal regions shift under climate change

Maberly et al.

Supplementary Table 1 RGB colour code for the nine thermal regions

Thermal region name	Name code	RGB colour code
Northern Frigid	NF	102, 102, 102
Northern Cool	NC	69, 117, 180
Northern Temperate	NT	116, 173, 209
Northern Warm	NW	255, 217, 47
Northern Hot	NH	255, 127, 0
Tropical Hot	TH	228, 26, 28
Southern Hot	SH	231, 138, 195
Southern Warm	SW	153, 112, 171
Southern Temperate	ST	84, 39, 136

Supplementary Table 2 description of the categories on the x-axes of Fig. 3 panels a and b.

**Code**	**Description**
**Panel a: Terrestrial ecoregions of the world**	
Tundra	Tundra
MontGrass	Montane Grasslands and Shrublands
Boreal/ Taiga	Boreal Forests/ Taiga
TempGras	Temperate Grasslands, Savannas, and Shrublands
TempCon	Temperate Coniferous Forests
TempBroad	Temperate Broadleaf and Mixed Forests
Med	Mediterranean Forests, Woodlands, and Scrub
Deserts	Deserts and Xeric Shrublands
TropMoist	Tropical and Subtropical Moist Broadleaf Forests
TropDry	Tropical and Subtropical Dry Broadleaf Forests
TropGrass	Tropical and Subtropical Grasslands, Savannas, and Shrublands
Flooded	Flooded Grasslands, Savannas., and Shrublands
Other	Two other categories not represented
**Panel b: Koppen-Geiger binomials**	
ET	Polar, Tundra
Ds	Cold, Dry summer
Dw	Cold, Dry winter
Df	Cold, Without dry season
Cw	Temperate, Dry winter
Cf	Temperate, Without dry season
BW	Arid, Desert
BS	Arid, Steppe
Af	Tropical, Rainforest
Am	Tropical, Monsoon
Aw	Tropical, Savannah

Supplementary Table 3 Location, characteristics, data extent and thermal regions for small lakes used in the analysis

Country	Site	Latitude	Longitude	Area (km^2)	No. data points	Start year	End year	Thermal region based on location	Thermal region based on *in situ* data	Data provider
Argentina	Caviahue North Arm	-37.87	-71.08	5.4	37	1999	2017	ST	SW	Monica Diaz
Argentina	Caviahue South Arm	-37.87	-71.08	3.8	35	2000	2017	ST	SW	Monica Diaz
Argentina	Lago Escondido	-41.05	-71.34	0.08	26	1987	1988	SW	SW	Monica Diaz
Argentina	Laguna Verde	-40.77	-71.65	0.0114	23	1989	1991	SW	SW	Monica Diaz
Belarus	Lake Batorina	54.9	26.7	6.3	186	1995	2011	NC	NC	Boris Adamovich
Belarus	Lake Myastro	54.9	26.7	13.1	184	1995	2011	NT	NC	Boris Adamovich
Canada	L224	49.69	-93.72	0.26	2375	1999	2011	NT	NT	Michael Paterson
Canada	L239	49.7	-93.7	0.54	3179	1996	2011	NT	NT	Michael Paterson
Canada	L240	49.7	-93.7	0.44	434	1996	2006	NT	NT	Michael Paterson
Canada	L260	49.7	-93.9	0.33	725	1999	2001	NT	NT	Michael Paterson
Canada	L373	49.7	-93.8	0.27	2358	2000	2001	NF	NT	Michael Paterson
Canada	L375	49.7	-93.8	0.53	330	1999	2002	NC	NC	Michael Paterson
Canada	L442	49.8	-93.8	0.16	2412	1999	2012	NT	NT	Michael Paterson
Canada	L625	49.8	-93.8	0.78	324	1999	2012	NT	NT	Michael Paterson
Canada	L626	49.8	-93.8	0.29	351	1999	2010	NT	NT	Michael Paterson
Canada	L658	49.7	-93.7	0.08	404	1999	2006	NT	NT	Michael Paterson
Canada	Blue Chalk	45.1999	-78.9432	0.5235	181	1996	2011	NT	NT	Andrew Paterson
Canada	Chubb Lake	45.2138	-78.9836	0.3441	183	1996	2011	NT	NT	Andrew Paterson
Canada	Crosson Lake	45.084	-79.036	0.5674	181	1996	2011	NT	NT	Andrew Paterson
Canada	Dickie Lake	45.151	-79.0876	0.936	261	1996	2011	NT	NT	Andrew Paterson
Canada	Harp lake	45.3798	-79.1335	0.7138	269	1996	2011	NT	NT	Andrew Paterson
Canada	Heney Lake	45.128	-79.103	0.2137	184	1996	2011	NT	NT	Andrew Paterson

Canada	Plastic Lake	45.1801	-78.8235	0.3214	260	1996	2011	NT	NT	Andrew Paterson
Canada	Red Chalk Main	45.19	-78.9486	0.4408	185	1996	2011	NT	NT	Andrew Paterson
China	Dongfeng	26.88	105.87	19.7	26	2011	2012	NW	NH	Baoli Wang
China	Wujiangdu	26.85	106.17	47.5	26	2011	2012	NH	NH	Baoli Wang
China	Bao-an	29.3	114	40	44	2001	2011	NH	NH	Hai-Jun Wang
Florida	Conway	28.47	-81.35	4.35	507	2011	2012	NH	NH	Mark Hoyer
Florida	Cherry	30.61	-83.41	1.94	342	2011	2014	NH	NH	Mark Hoyer
Florida	Dias	29.16	-81.32	2.88	354	2011	2012	NH	NH	Mark Hoyer
Florida	Dora	28.79	-81.69	18.11	515	2011	2012	NH	NH	Mark Hoyer
Florida	Dorr	29.002	-81.622	7.59	606	2011	2012	NH	NH	Mark Hoyer
Florida	Eloise	27.9833	-81.7067	4.69	387	2011	2012	NH	NH	Mark Hoyer
Florida	Grasshopper	29.136	-81.614	0.47	515	2011	2012	NH	NH	Mark Hoyer
Florida	Halfmoon	28.92	-82.265	0.36	546	2011	2013	NH	NH	Mark Hoyer
Florida	Monroe	81.27	-28.84	38.07	606	2011	2012	NH	NH	Mark Hoyer
Florida	Panasoffkee	28.804	-82.122	18.05	431	2011	2012	NH	NH	Mark Hoyer
Florida	Sampson	29.928	-82.19	7.55	494	2011	2012	NH	NH	Mark Hoyer
Florida	Santa Fe	29.74	-82.0771	20.11	494	2011	2012	NH	NH	Mark Hoyer
Florida	Starke	28.57	-81.5367	1.33	506	2011	2012	NH	NH	Mark Hoyer
Florida	Stick Marsh	27.8067	-80.72	9.63	476	2011	2012	NH	NH	Mark Hoyer
Florida	Trafford	26.42	-81.4925	6.05	381	2011	2012	NH	NH	Mark Hoyer
Florida	Washington	28.145	-80.58	17.65	476	2011	2012	NH	NH	Mark Hoyer
Florida	Weir	29.02	-81.9375	28.62	485	2011	2012	NH	NH	Mark Hoyer
Florida	Wildcat	29.16	-81.63	1.42	576	2011	2012	NH	NH	Mark Hoyer
Greenland	Site 6	67	-51.11	0.215	92	1999	1999	NF	NF	John Anderson & Helen Kettle
Greenland	Site 27	66.82	-51.64	0.073	91	1999	1999	NF	NF	John Anderson & Helen Kettle
Greenland	Site 28	66.83	-51.63	0.037	94	1999	1999	NF	NF	John Anderson & Helen Kettle

Greenland	Site 42	66.74	-51.81	0.067	92	1999	1999	NF	NF	John Anderson & Helen Kettle
Greenland	Site 43	66.73	-51.81	0.095	92	1999	1999	NF	NF	John Anderson & Helen Kettle
Iceland	Bakkatjorn Pond	64.2	-22	0.06	59	2007	2011	NC	NF	Thora Hrafnsdottir
Iceland	Ellidavatn	64.1	-21.8	1.8	136	2002	2011	NC	NF	Thora Hrafnsdottir
Iceland	Hafravatn	64.1	-21.7	1	117	2004	2011	NC	NF	Thora Hrafnsdottir
Iceland	Raudavatn	64.1	-21.8	0.33	115	2004	2011	NC	NF	Thora Hrafnsdottir
Iceland	Reykjvikurtjohn	64.1	-21.9	0.1	54	2008	2011	NC	NF	Thora Hrafnsdottir
Iceland	Mynvatn	65.6	-17	29	1572	2007	2011	NC	NC	Arni Einarsson
Iceland	Vifilsstadavatn	64.1	-21.9	0.27	118	2004	2011	NC	NF	Thora Hrafnsdottir
New Zealand	Rotoehu	-38	176.5	8	174	1995	2011	SW	SW	David Hamilton
New Zealand	Rotoiti	-38.1	176.4	34	192	1995	2011	SW	SW	David Hamilton
New Zealand	Rotoma	-38	176.6	11.2	132	1995	2011	SW	SW	David Hamilton
Papua New Guinea	Waigani	-9.4	147.2	1.2	20	1980	1981	TH	TH	Osborne (1991) *Int. Rev. Ges Hydrobiol.* **76**, 105-120.
Spain	Albufera des Grau	39.56	-4	0.78	94	2002	2007	NW	NW	Biel Obrador

UK	Bassenthwaite Lake	54.65	-3.22	5.3	421	1995	2011	NT	NT	UKCEH
UK	Blelham Tarn	54.4	-3	0.1	421	1995	2011	NW	NT	UKCEH
UK	Derwent Water	54.6	-3.1	5.4	424	1995	201	NT	NT	UKCEH
UK	Esthwaite Water	54.4	-3	1	444	1995	2011	NT	NT	UKCEH
UK	Loweswater	54.6	-3.4	0.6	69	2000	2010	NT	NT	UKCEH
UK	Grasmere	54.4	-3	0.6	430	1995	2011	NT	NT	UKCEH
UK	Windermere North	54.4	-3	8.1	444	1995	2011	NW	NT	UKCEH
UK	Windermere South	54.3	-3	6.7	442	1995	2011	NW	NT	UKCEH
UK	Fionnaraich	57.5	-5.4	0.09	731	2007	2009	NT	NT	Simon Patrick/ ENSIS

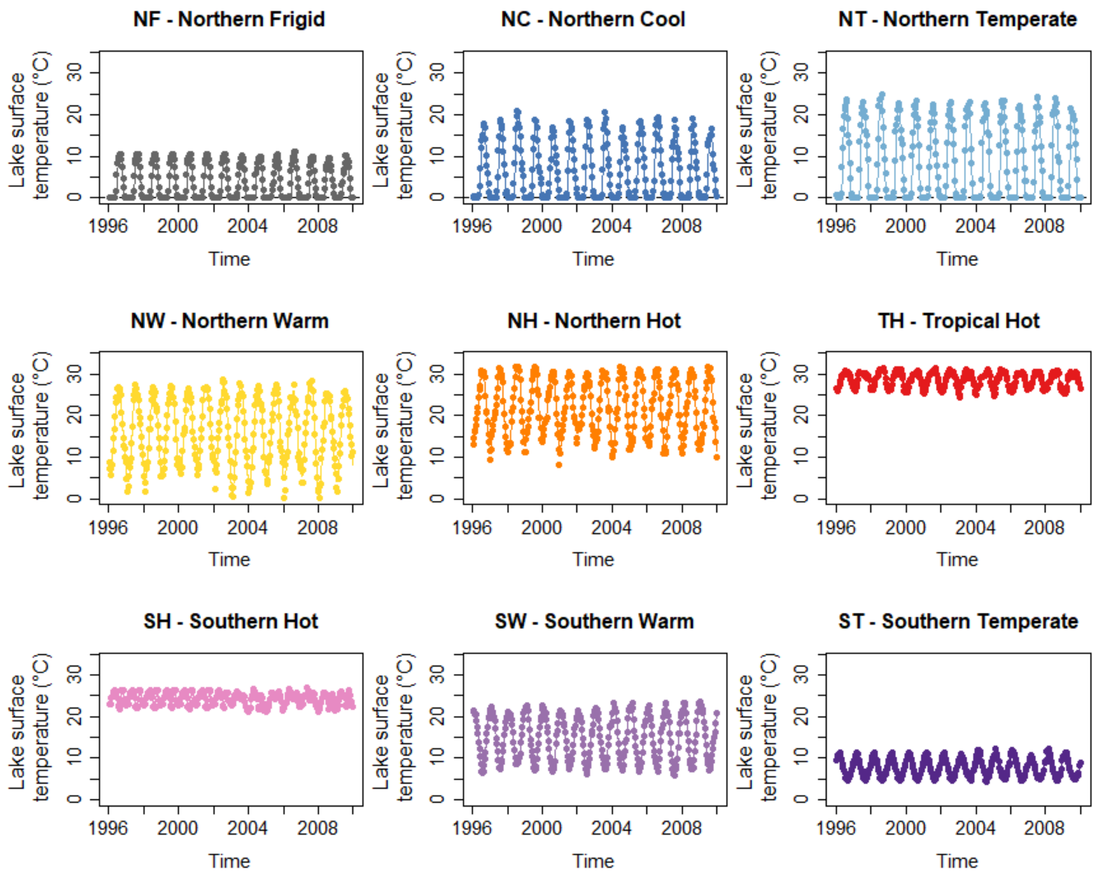

Supplementary Fig. 1 Seasonal dynamics of the median lake for each lake thermal region. The symbols represent the satellite ARC-Lake data from 1996 to 2011, the line is the smooth curve. The median lake was selected on the basis of the annual mean surface temperature.

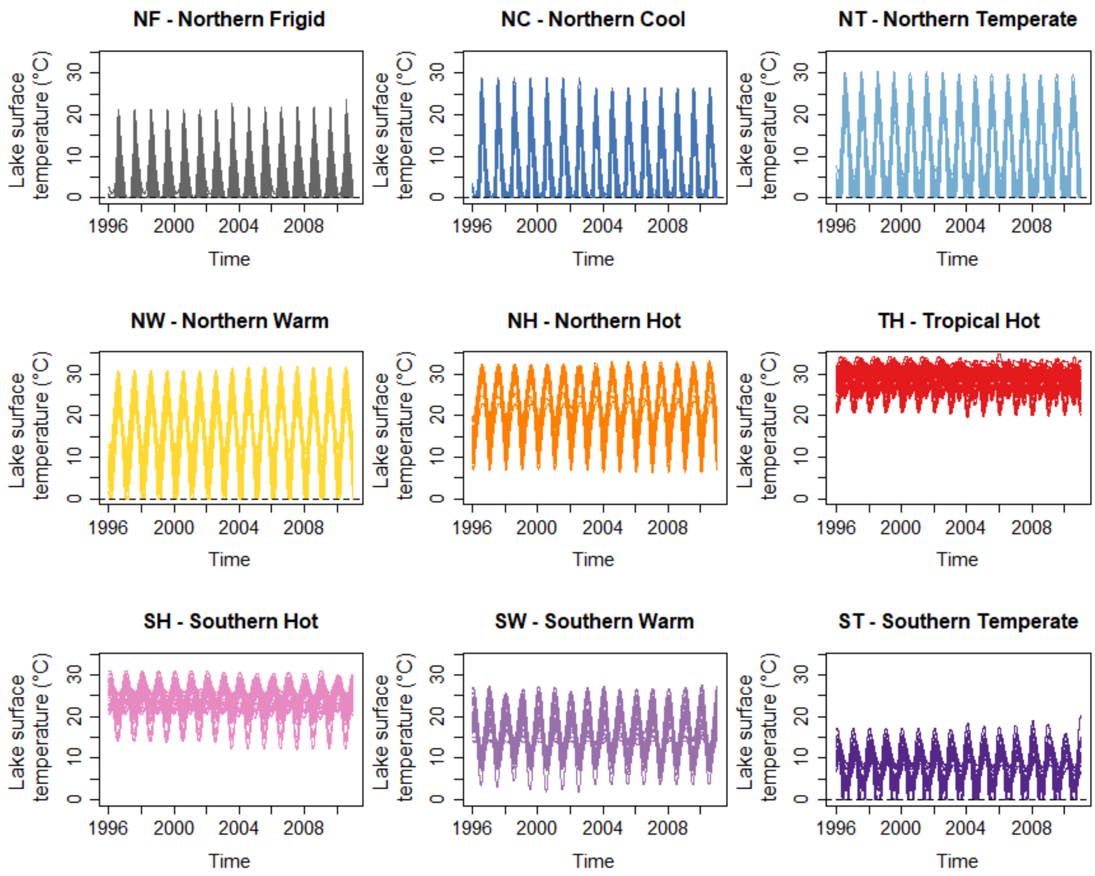

Supplementary Fig. 2 Smooth curves for all the 732 lakes allocated to thermal regions. The satellite ARC-Lake data from 1996 to 2011 show synchronous patterns of lake time series within a group.

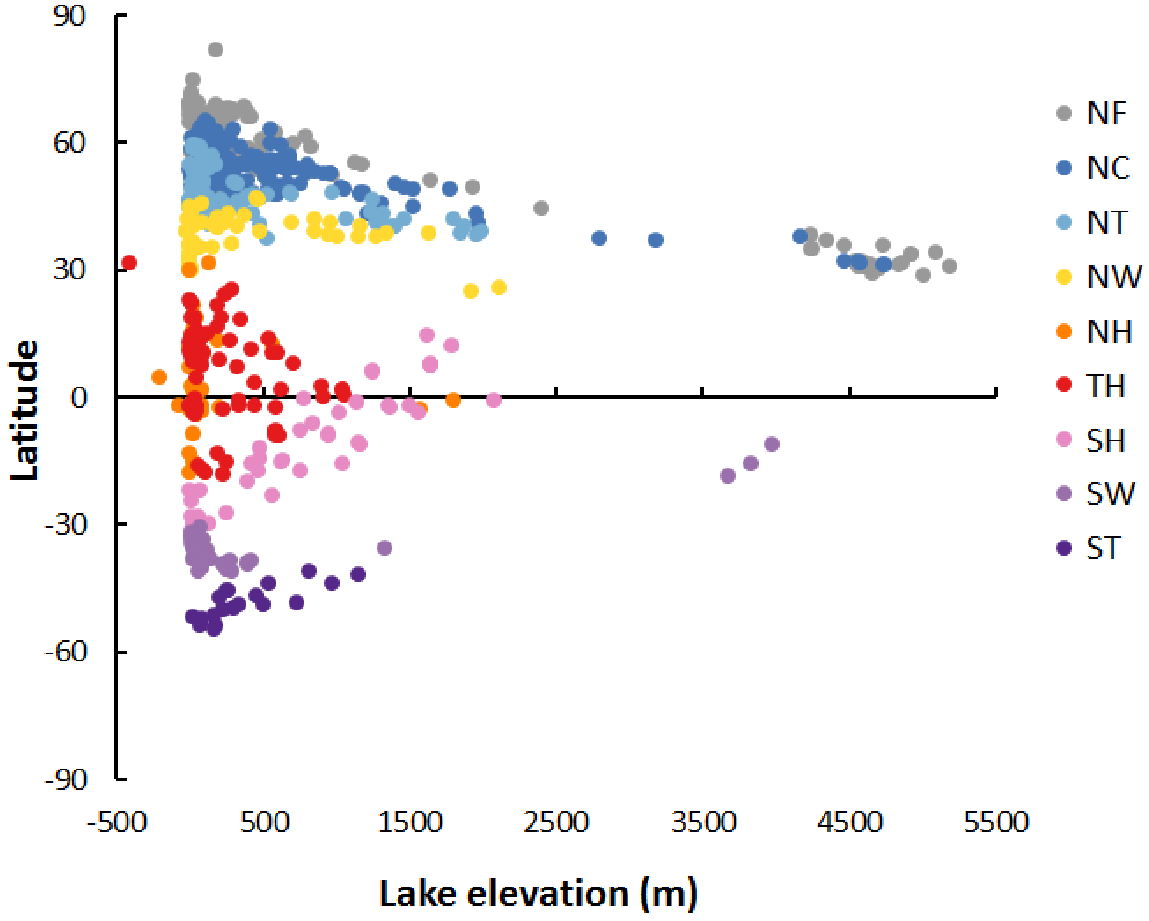

Supplementary Fig. 3 Effect of elevation on the latitude at which each thermal region occurs. For a given lake thermal region, lakes at high elevation occur closer to the equator than lakes at low elevation.

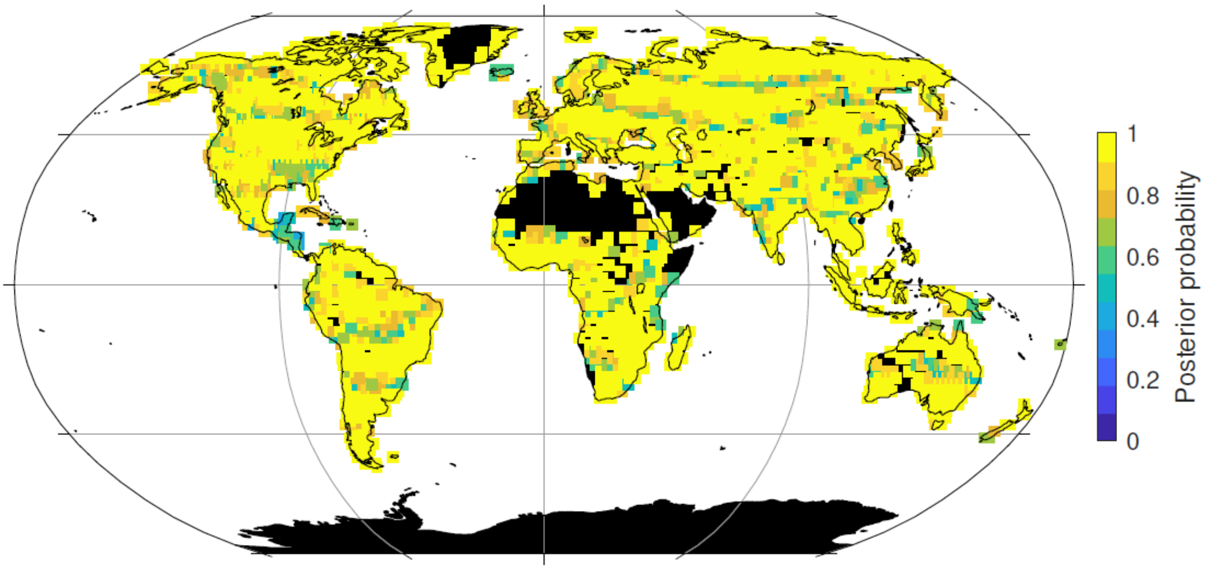

Supplementary Fig. 4 Posterior probability of thermal region membership. The magnitude of classification uncertainty is represented by the posterior probability of class membership corresponding to the thermal region it belongs to as predicted by quadratic discriminant analysis (QDA; see Methods).

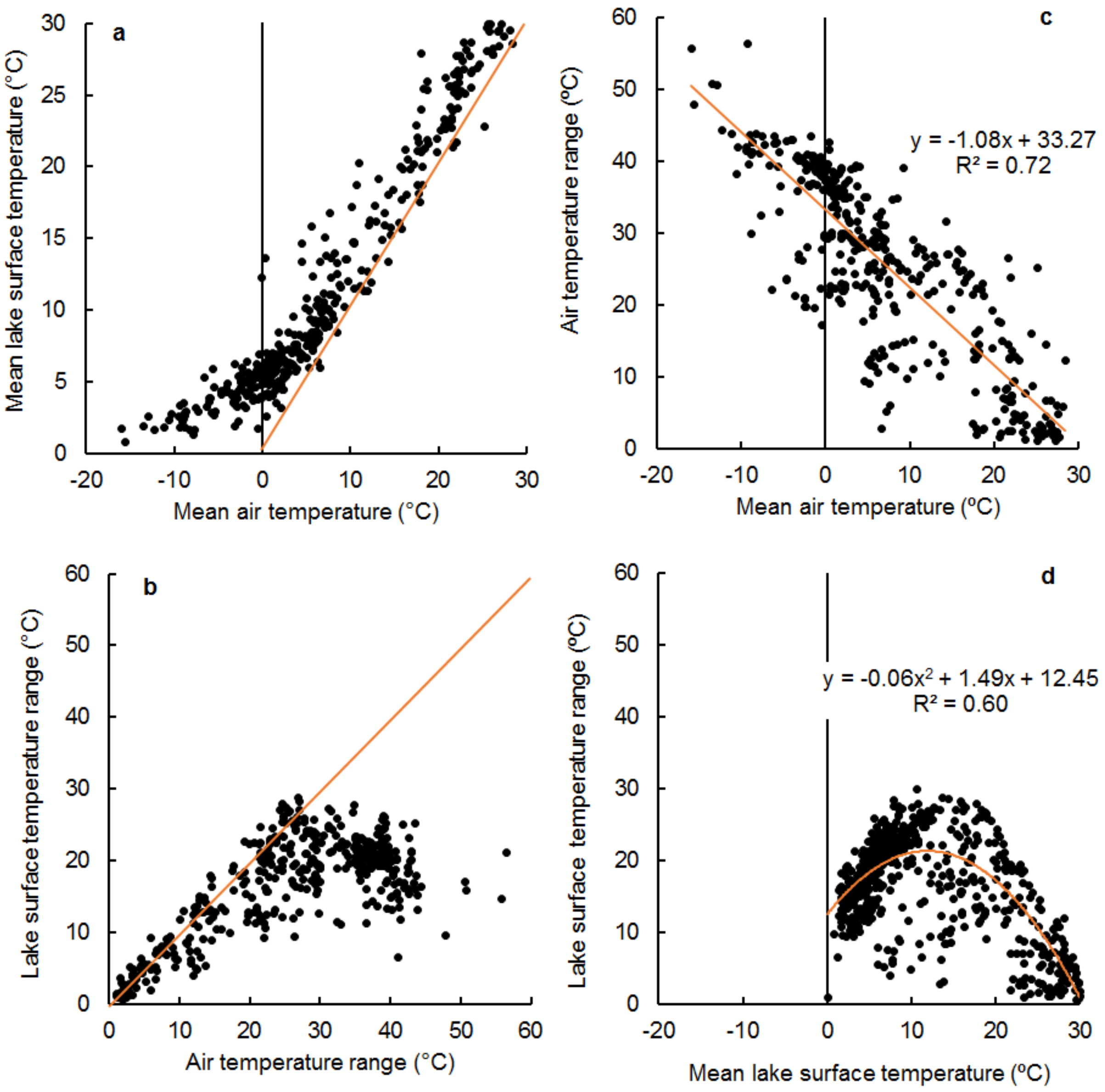

Supplementary Fig. 5 Comparison of lake and air temperature dynamics from the 732 ARCLakes. **a**, Mean temperature of lake surface vs air. **b**, Temperature range of lake surface vs air. **c**, Air temperature range vs mean air temperature. **d**. Lake surface temperature range vs mean lake surface temperature. The orange lines are the 1:1 line in **a** and **b**, a linear regression in **c** and a 2nd order polynomial regression in **d**.

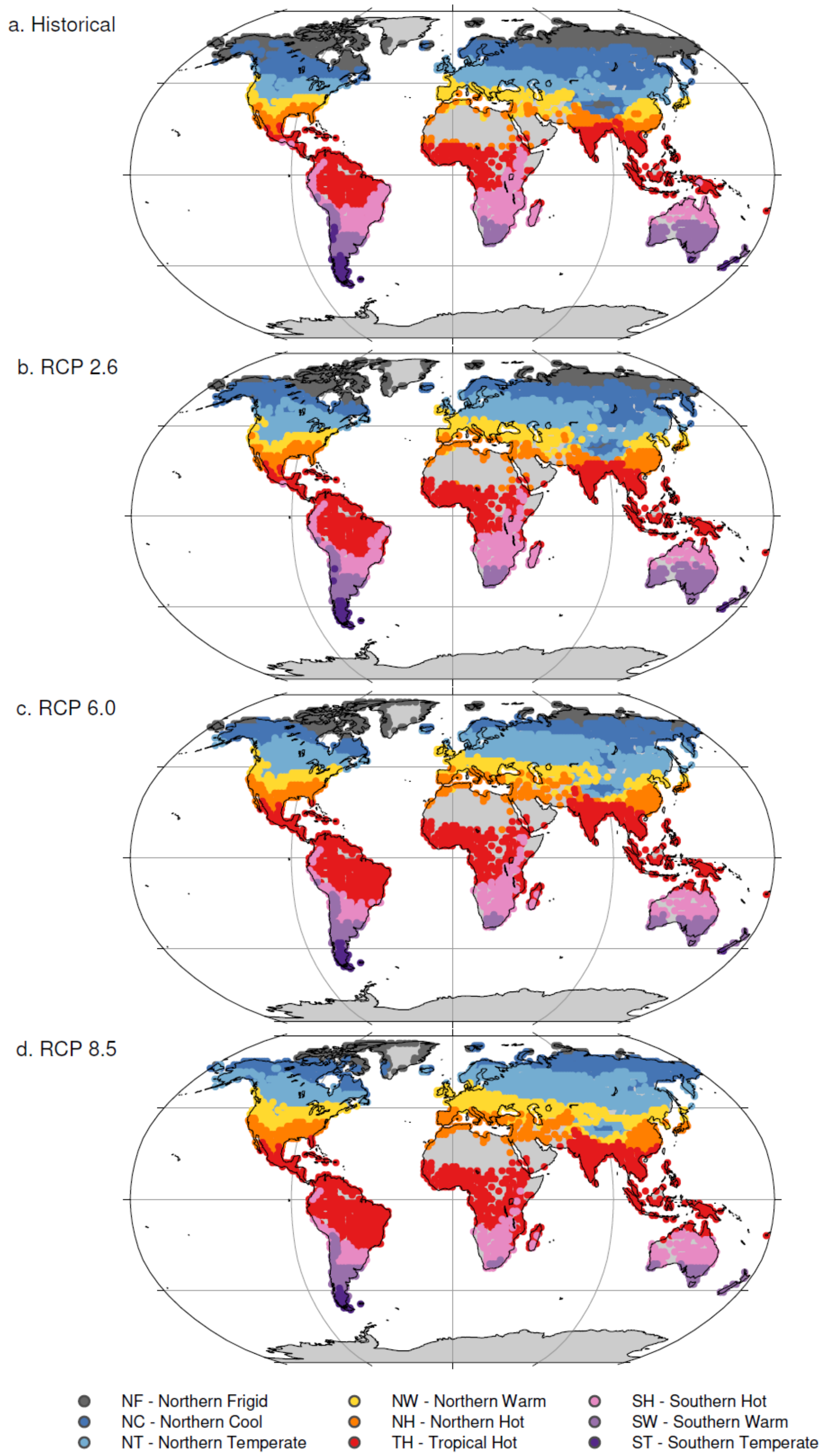

Supplementary Fig. 6 Global distribution of lake thermal regions forced with bias-correctedHadGEM2-ES projections. a, historic, **b**, RCP 2.6, **c**, RCP 6.0 and **d**, RCP 8.5. Grey represents areas without lakes.

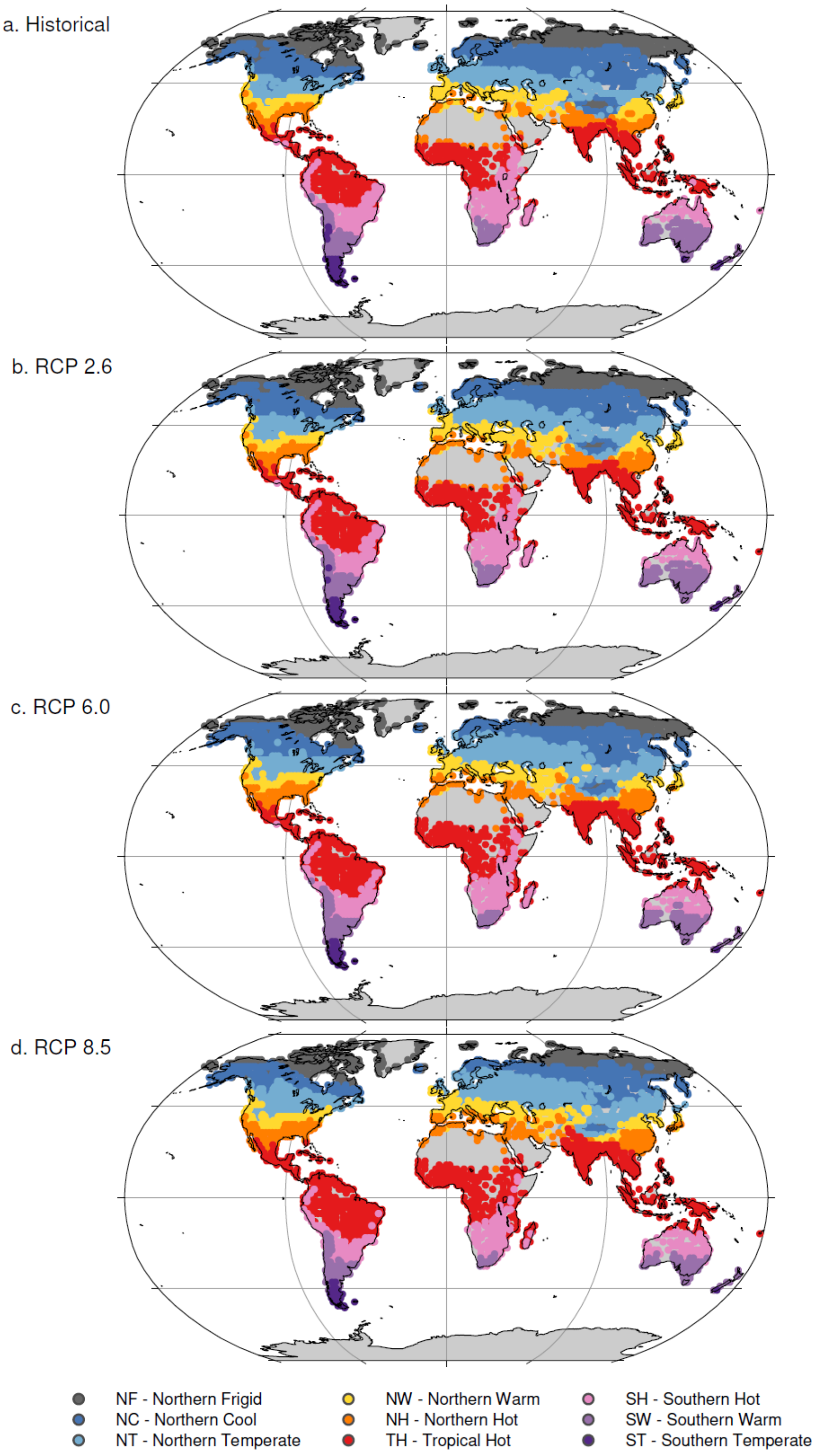

Supplementary Fig. 7 Global distribution of lake thermal regions forced with bias-corrected GFDL-ESM2M projections. **a**, historic, **b**, RCP 2.6, **c**, RCP 6.0 and **d**, RCP 8.5. Grey represents areas without lakes.

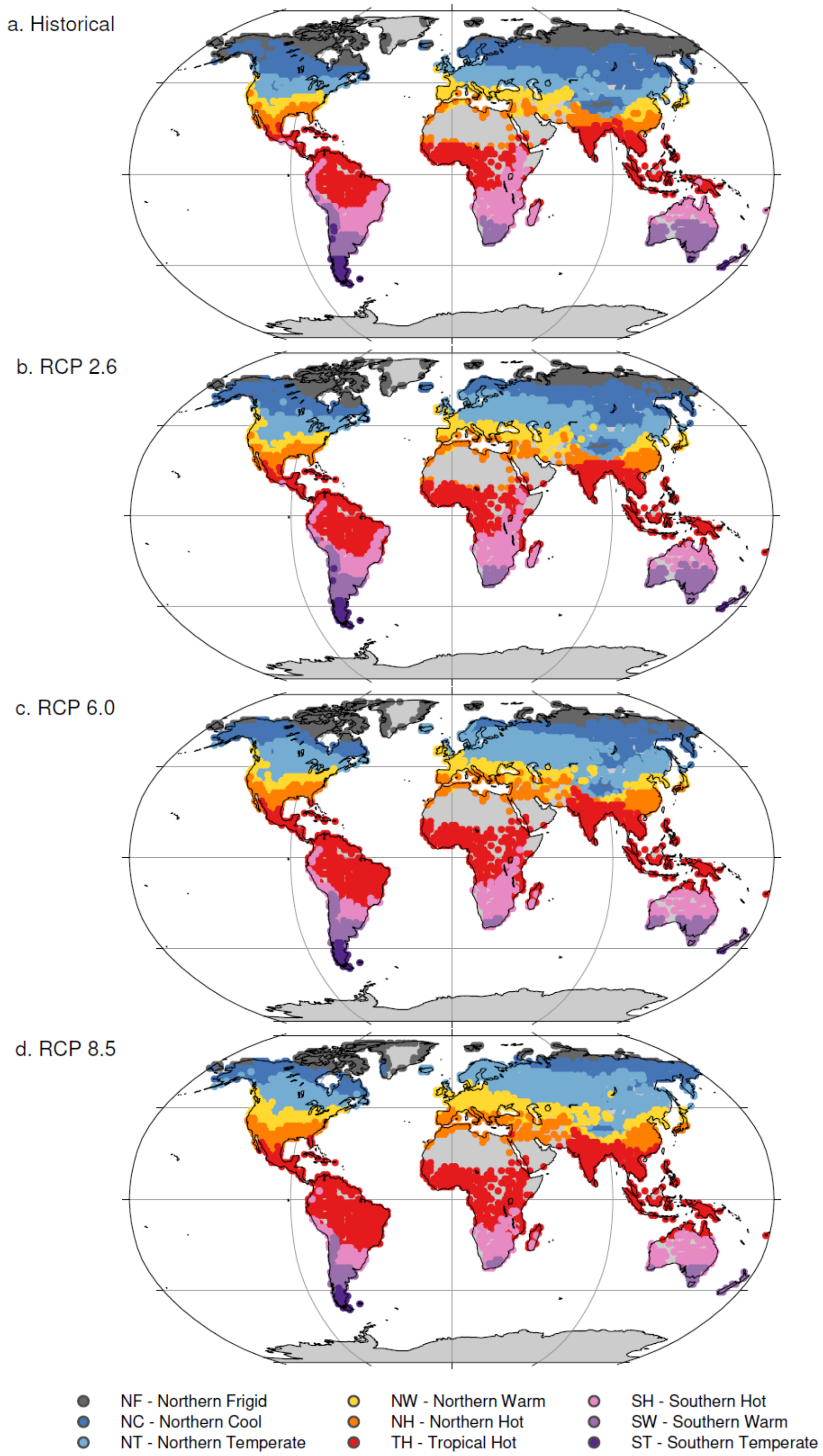

Supplementary Fig. 8 Global distribution of lake thermal regions forced with bias-corrected IPSL-CM5A-LR projections. **a**, historic, **b**, RCP 2.6, **c**, RCP 6.0 and **d**, RCP 8.5. Grey represents areas without lakes.

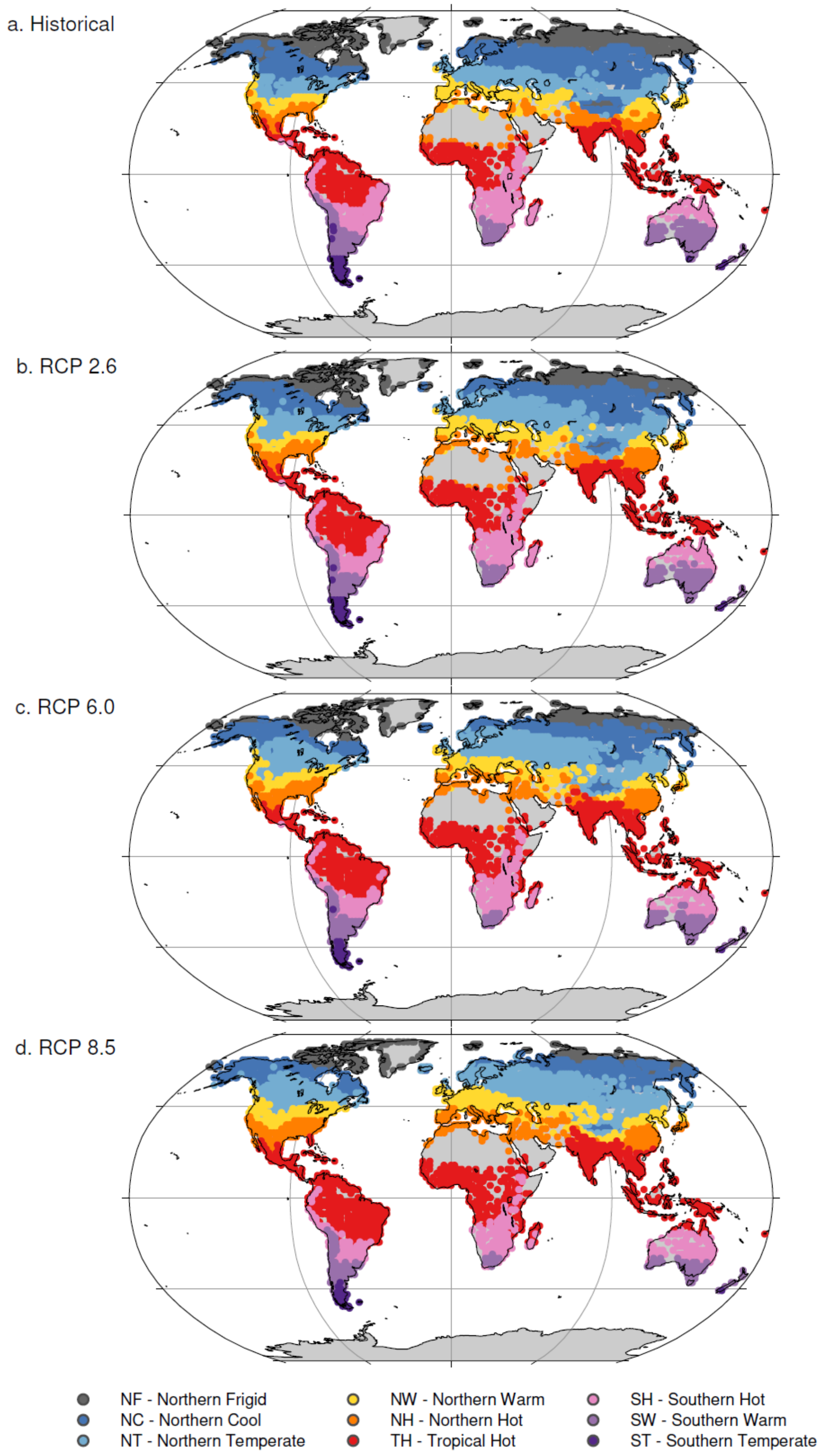

Supplementary Fig. 9 Global distribution of lake thermal regions forced with bias-corrected MIROC5 projections. **a**, historic, **b**, RCP 2.6, **c**, RCP 6.0 and **d**, RCP 8.5. Grey represents areas without lakes.

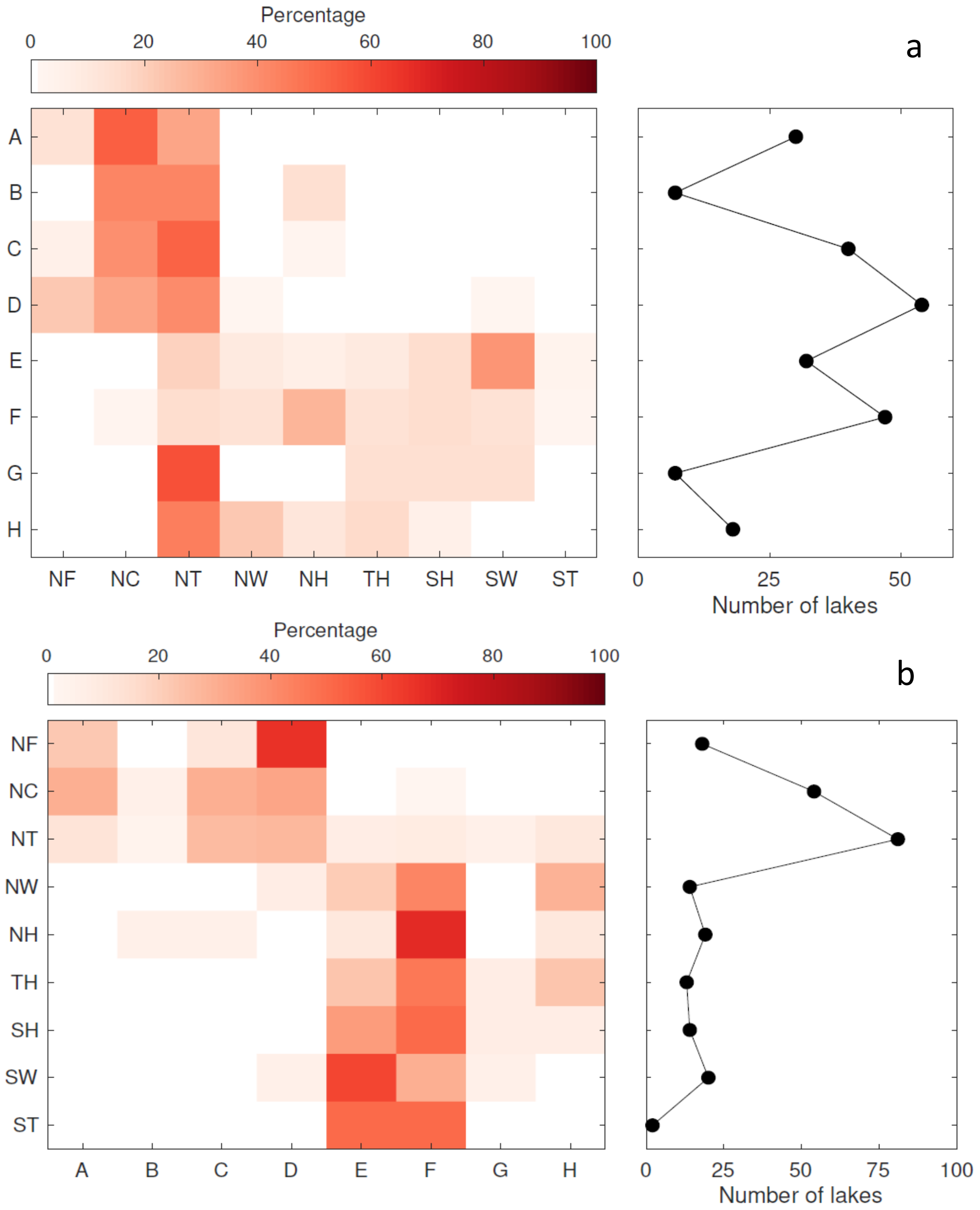

Supplementary Fig. 10. **Comparison of allocation between published regression tree clusters and lake thermal regions.** The 235 lakes in the regression tree leaf clusters in O'Reilly et al[1] (designated A to H) are compared to the lake thermal regions. **a**. the % of lakes in a regression tree cluster in each thermal regions; **b**. the % of lakes in a thermal region in each regression tree cluster. The right hand scatter plots show the number of lakes that make up the percentage for each cluster **a** or thermal region **b**.

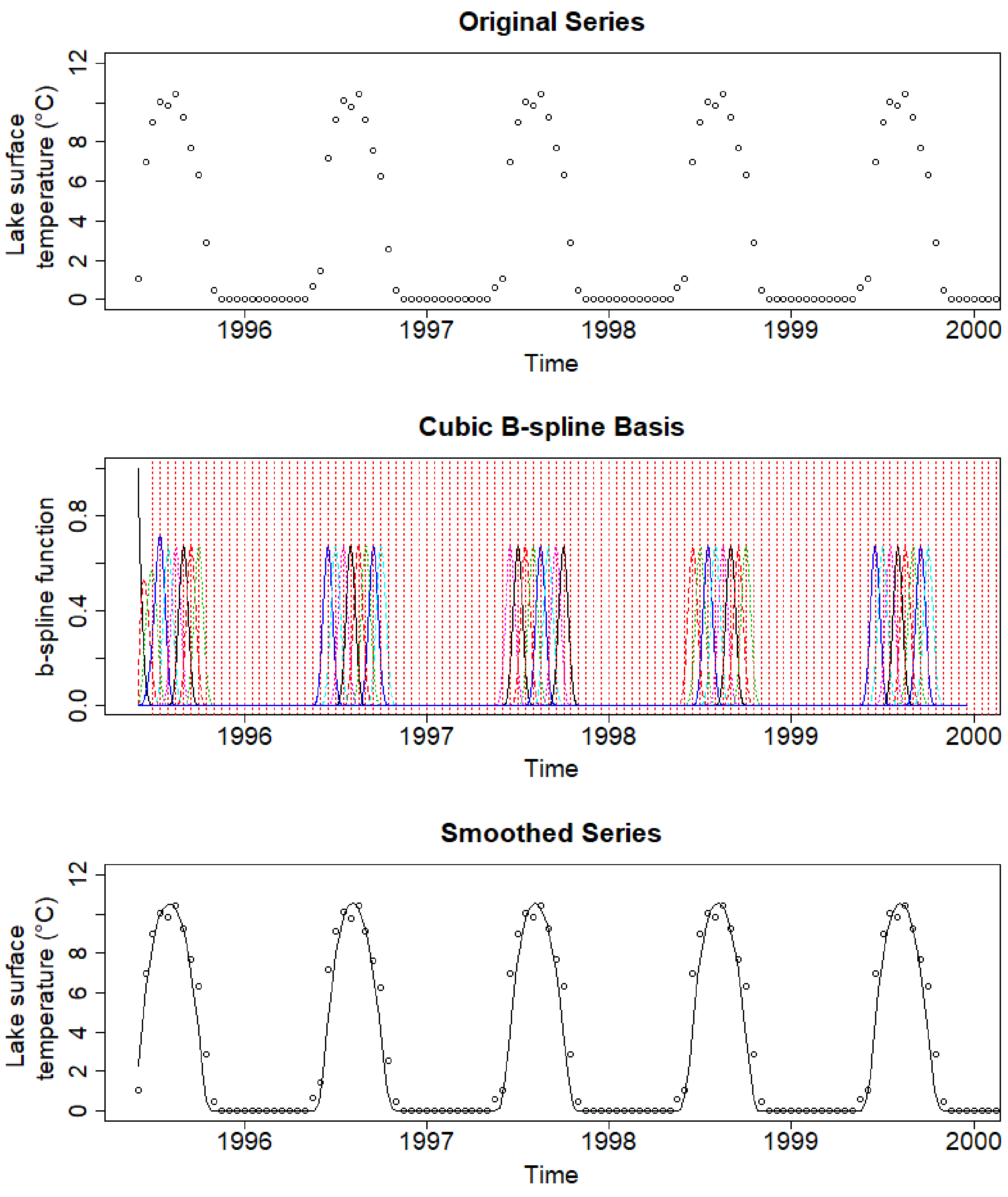

Supplementary Fig. 11 Example conversion of an original time series of lake surface water temperature to a smoothed time series using a cubic b-spline basis. The vertical red lines show the placement of the internal knots at each time point and the different curves represent the b-spline basis functions used for smoothing.

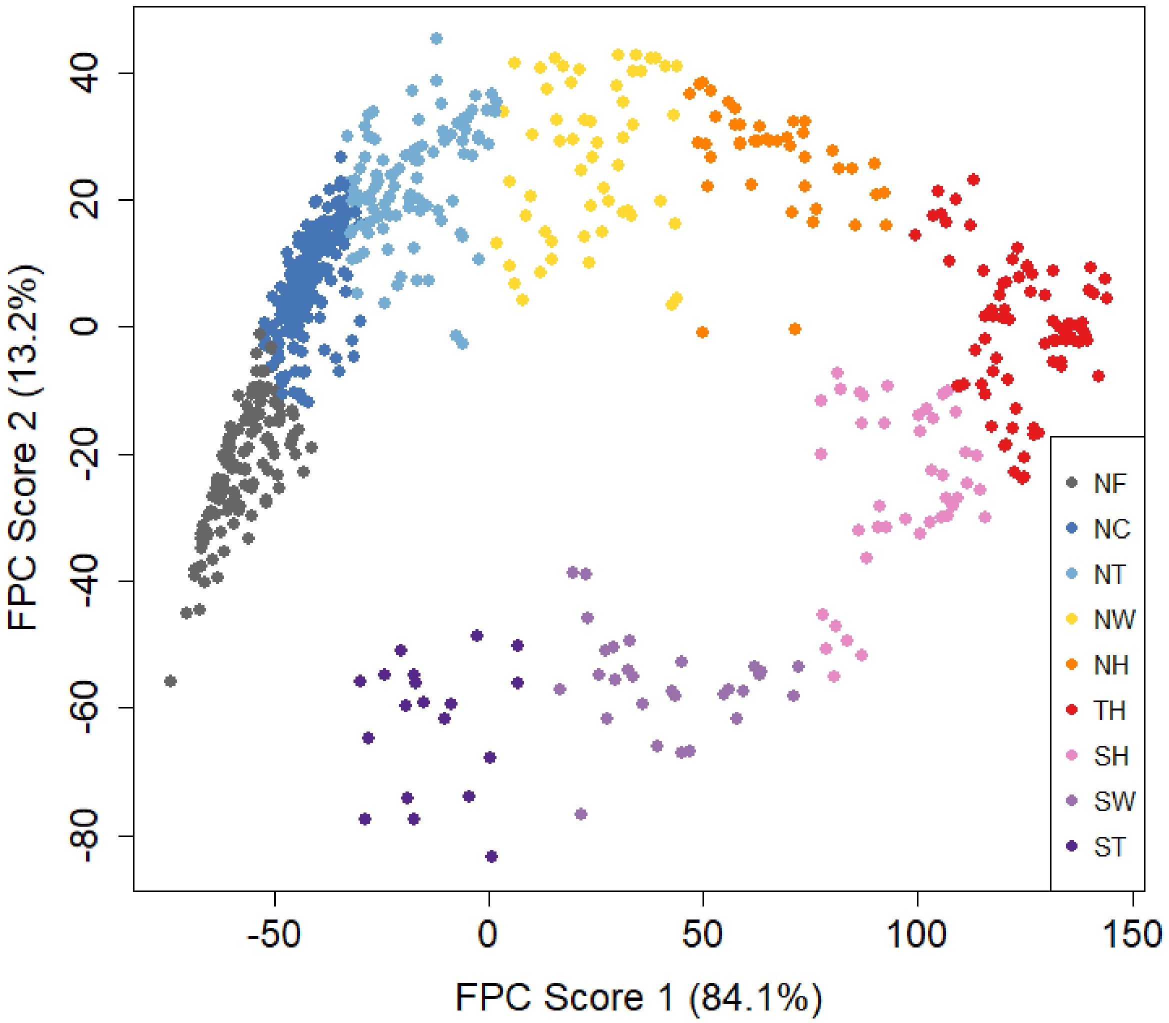

Supplementary Fig. 12 Functional principal components scores for the nine thermal regions. Supplementary Fig. 1. Gives the full names of the thermal regions.

Supplementary References

1. O'Reilly CM, *et al.* Rapid and highly variable warming of lake surface waters around the globe. *Geophysical Research Letters* **42**, 10773-10781 (2015).
